# Analgesic effects of NB001 on mouse models of arthralgia

**DOI:** 10.1186/s13041-015-0151-9

**Published:** 2015-10-09

**Authors:** Zhen Tian, Dong-sheng Wang, Xin-shang Wang, Jiao Tian, Jing Han, Yan-yan Guo, Bin Feng, Nan Zhang, Ming-gao Zhao, Shui-bing Liu

**Affiliations:** Department of Pharmacology, School of Pharmacy, Fourth Military Medical University, Xi’an, 710032 China; Department of Orthopedics, Jinling Hospital, Clinical School of Nanjing, Second Military Medical University, Nanjing, 210002 China; Department of Pediatrics, Tangdu Hospital, Fourth Military Medical University, Xi’an, 710038 China; Department of Pharmacy, School of Stomatology, Fourth Military Medical University, Xi’an, 710032 China

**Keywords:** Adenylyl cyclase 1, Arthritis, Pain, NB001, Inflammation

## Abstract

Our previous studies have demonstrated the critical roles of calcium-stimulated adenylyl cyclase 1 (AC1) in the central nervous system in chronic pain. In the present study, we examined the analgesic effects of NB001, a selective inhibitor of AC1, on animal models of ankle joint arthritis and knee joint arthritis induced by complete Freund’s adjuvant injection. NB001 treatment had no effect on joint edema, stiffness, and joint destruction. Furthermore, the treatment failed to attenuate the disease progression of arthritis. However, NB001 treatment (3 mg/kg) significantly weakened joint pain-related behavior in the mouse models of ankle joint arthritis and knee joint arthritis. Results indicated that NB001 exhibited an analgesic effect on the animal models of arthritis but was not caused by anti-inflammatory activities.

## Background

Arthritis is the most common musculoskeletal disease and is characterized by joint inflammation, stiffness, pain, and cartilage damage [1]. Among these symptoms, arthralgia negatively influences the articular function and quality of life of patients. Chronic arthralgia not only leads to mobility disorders and physical disability but also causes a substantial amount of emotional and economic stress [2, 3]. However, the mechanism underlying arthritic joint pain is still poorly understood. Non-steroidal anti-inflammatory drugs and opioids have been widely used as therapies in the clinical treatment of arthritic joint pain for decades [4, 5]. However, the long-term use of both treatments exhibit non-negligible adverse effects.

Arthritic joint pain often occurs when the affected joint is moved and/or loaded but can also occur at rest [6, 7]. Spontaneous pain (joint pain at rest), movement-evoked pain, thermal hyperalgesia, mechanical allodynia, and joint hyperalgesia are the main features of arthritic pain [8–10]. Arthritic joint pain results in part from inflammation-induced articular nerve sensation and the activation of peripheral and central neuronal mechanisms [11]. Upon development of inflammation, numerous silent joint nociceptors develop sensitivity for stimulation; this recruitment of fibers significantly increases input into the spinal cord [12, 13]. Ascending spinal nociceptive neurons show gradually increasing response to the innocuous and noxious stimulation exerted on inflamed joints. The generation and maintenance of central sensitization are mediated by various transmitter systems in the spinal cord and related brain regions [14]. As a key transmitter in the nervous system, glutamate, including its receptors, is essential for joint nociception; furthermore, NMDA receptor activation is critically involved in central sensitization and joint inflammatory hypersensitivity [15, 16].

Cyclic adenosine monophosphate (cAMP) is a key intracellular second messenger involved in many physiological functions, such as nociception perception, learning and memory, emotional fear, and addiction [17–19]. As an enzyme that catalyzes ATP to cAMP, calcium-stimulated adenylyl cyclase subtype 1 (AC1) is highly expressed in neurons and critically involved in pain-related long-term potentiation (LTP) in both the spinal cord dorsal horn [20] and the anterior cingulate cortex (ACC) [21]. AC1 significantly contributes to behavioral sensitization and spinal facilitation in animal models of pain as a downstream of glutamate NMDA receptors [22]. AC1 knockout mice have been known to show hypalgesia to inflammatory pain, muscle pain, and neuropathic pain without evident alterations in physiological functions [23–25]. NB001, a selective inhibitor of AC1, exhibits analgesic effects on several animal models of chronic pain, including neuropathic pain, inflammation pain, and irritable bowel syndrome-induced visceral pain [23, 26]. Furthermore, no obvious side effects have been observed when NB001 is applied in animal models [23].

The analgesic effects of NB001 on arthritic joint pain have not been investigated. In this study, we aimed to examine the analgesic effect of NB001 on the (i) ankle joint pain induced by injecting complete Freund’s adjuvant (CFA) into the hind metatarsal footpad, as well as on the (ii) knee joint pain caused by CFA administration into the knee joint cavity. The anti-inflammatory effects of NB001 on CFA-induced arthritis, including edema, stiffness, and joint damage were also measured.

## Results

### NB001 exerted no effect on CFA-induced joint inflammation

**Fig. 1:**
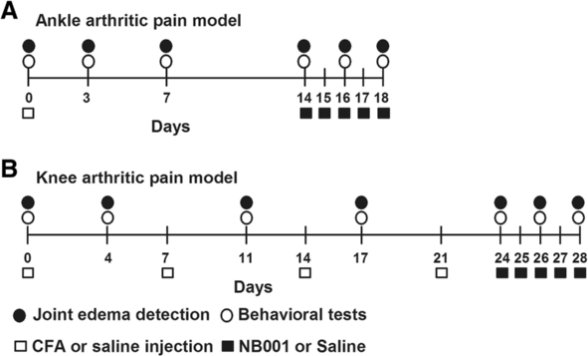
Schematic experimental procedure of two arthritic pain models. On the day before CFA injection (day 0), basal behavioral tests and joint diameters were detected. Then, mice were injected with CFA in hind metatarsal footpad (upper paradigm) or into intra-articular space of knee (lower paradigm) to induce arthritic pain of ankle and knee respectively. For the knee arthritis model, the mice received another three intra-articular CFA injection on day 7, 14, 21. For the control mice, the same volume of saline was injected at the same time points with that of CFA injection. Behavioral tests were performed on day 3, 7, 14 16, 18 for ankle arthritic pain model and day 4, 11, 17, 24, 26, 28 for knee arthritic pain model. NB001 (3 mg/kg, i.p.) or saline was injected to mice once daily from day 14 to day 18 in ankle arthritis model and day 24 to day 28 in knee arthritis model. At the first day of treatment (day 14 and day 24 respectively), NB001 or saline was administrated after the behavioral tests. On day 15, 17 in ankle arthritis model and day 25, 27 in knee arthritis model, the mice were just received NB001 or saline administration and no behavioral tests were performed. The related behavioral tests were conducted within 2.5 h on day 16, 18 and day 26, 28 in two models respectively following NB001 or saline treatment

**Fig. 2:**
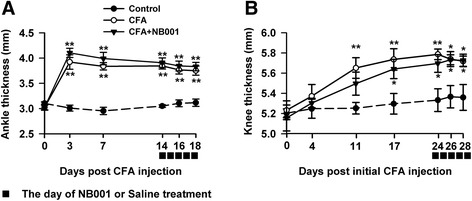
The effect of NB001 on joints edema induced by CFA injection. **a** Ankle diameter reached the maximum on day 3 following CFA injection and then it remained at a higher level compared with control. NB001 treatment (3 mg/kg, i.p.) did not attenuate the ankle edema. **b** There was a significant increase of knee diameter after intra-articular CFA injection. NB001 administration did not alleviate the inflammation. *n* = 8 mice in each group. ^*^ *p* < 0.05, ^**^ *p* < 0.01 compared to the control group

Experimental procedure of two arthritic pain models was sown in the Fig. 1. To assess joint inflammation quantitatively, the diameters of the ankle joint or knee joint were measured by using a vernier caliper. Figure 2a shows inevitable ankle joint edema 3 days after the mice received CFA injection in the hind metatarsal footpad. The edema lasted at least to the end of the experiment (day 18). NB001 administration (3 mg/kg, from day 14 to day 18) showed no obvious effects on ankle edema. In the knee joint model, the joint thickness of the knee increased gradually after the initial CFA injection. Similarly, NB001 treatment cannot alleviate knee joint swelling (Fig. 2b). Results showed that NB001 may exhibit no effects on CFA-induced joint inflammation.

### Effect of NB001 on joint pain-related behaviors

**Fig. 3:**
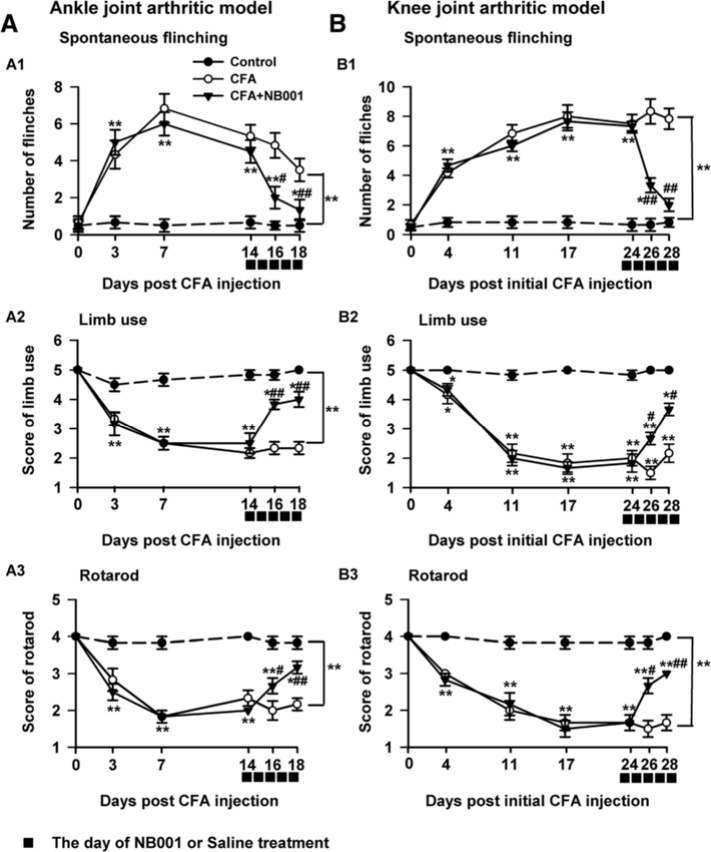
NB001 alleviates arthritic pain behaviors. **a** Data from the ankle joint arthritic model. *A1*: The number of spontaneous flinches increased significantly and reached a peak on day 7 following CFA injection and maintained at a higher level as compared with the control. In addition, the score of normal limb use (*A2*) and forced ambulation (rotarod) (*A3*) declined significantly after the CFA injection. **b** Data from the knee joint arthritic model. *B1*: Mice exhibited a gradually increased spontaneous flinches. The score of normal limb use (*B2*) and forced ambulation (rotarod) (*B3*) declined significantly after the CFA injection. NB001 treatment (3 mg/kg, i.p.) significantly attenuated pain-related behaviors in two types of model (**a** and **b**). *n* = 8 in each group. ^*^ *p* < 0.05, ^**^ *p* < 0.01 compared to the control group; ^#^ *p* < 0.05, ^##^ *p* < 0.01 compared to the CFA group

Flinching behavior indicates spontaneous pain, which mimics the clinical condition of arthritic patients with joint pain at rest. Subsequent to CFA injection into the metatarsal footpad, the number of hindpaw flinches significantly increased, peaked on day 7, and then decreased gradually but remained apparently higher than that of control mice (Fig. 3a1). For knee joint arthritic models, the number of flinches markedly increased and peaked on day 17 compared with the control (Fig. 3b1). NB001 treatment (3 mg/kg) sharply decreased the number of flinches in both ankle and knee arthritic animal models. On day 28, the number of flinches of the knee joint arthritic mice treated with NB001 became comparable with that of normal mice (Fig. 3b1).

Limb use, an indicator of stimulus-evoked pain, was measured (with scores ranging from five to zero) to further evaluate the effects of NB001. The mean score of limb use rapidly decreased after the mice received CFA injection either into the hind metatarsal footpad (Fig. 3a2) or the knee joint cavity (Fig. 3b2). NB001 administration for 5 days significantly increased the limb use score in mice with ankle and knee joint arthritis.

Subsequently, limb use during forced ambulation was detected by using a rotarod test with a similar scale. Figure 3a3, b3 show a remarkable decrease in the average score of forced limb use in both types of arthritic models. NB001 administration obviously relieved pain, as reflected by the rotarod test in two animal models. These combined results revealed persistent joint pain in CFA-induced ankle arthritis or knee joint arthritis. Furthermore, NB001 showed evident analgesic effects on adjuvant-induced joint pain.

### NB001 increased the mechanical threshold

**Fig. 4:**
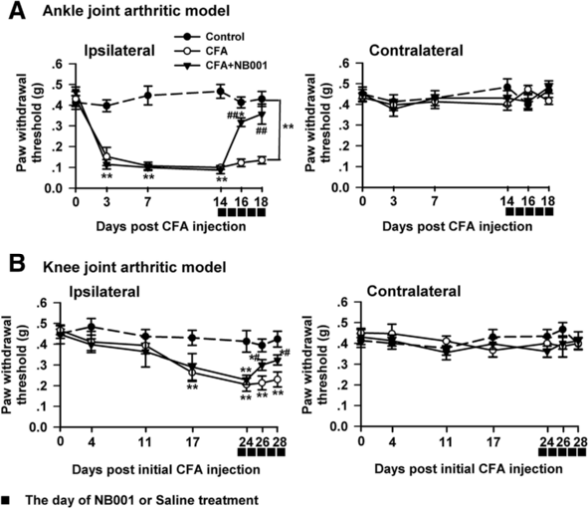
NB001 attenuates mechanical allodynia. **a** Mechanical threshold in the ipsilateral hindpaws decreased in the mice following CFA injection into metatarsal footpad, but had no alteration in the contralateral paws. NB001 administration attenuated the mechanical allodynia in the ipsilateral hindpaws. **b** The mechanical allodynia occurred gradually following knee intra-articular CFA injection and the threshold decreased obviously from day 17 after initial CFA injection (three days after the third CFA injection), but had no alteration in the contralateral paws. NB001 administration attenuated the mechanical allodynia in the ipsilateral hindpaws. *n* = 8 mice in each group. ^*^ *p* < 0.05, ^**^ *p* < 0.01 compared to the control group; ^#^ *p* < 0.05, ^##^ *p* < 0.01 compared to the CFA group

To further examine the analgesic effect of NB001 on the types of pain behavior associated with arthritis, mechanical allodynia was detected in both animal models. The mechanical paw withdrawal threshold (MPWT) in ipsilateral hindpaw decreased immediately and significantly in mice injected with CFA into the metatarsal footpad (Fig. 4a). NB001 administration (3 mg/kg from day 14) markedly increased the mechanical threshold on days 16 and 18 compared with saline treatment (Fig. 4a). Knee intra-articular CFA injection gradually decreased MPWT and NB001 obviously attenuated mechanical allodynia (Fig. 4b). No obvious difference was observed in MPWT in contralateral paws among groups in both types of animal models (Fig. 4a, b) within the entire experimental period.

### NB001 increased the thermal threshold

**Fig. 5:**
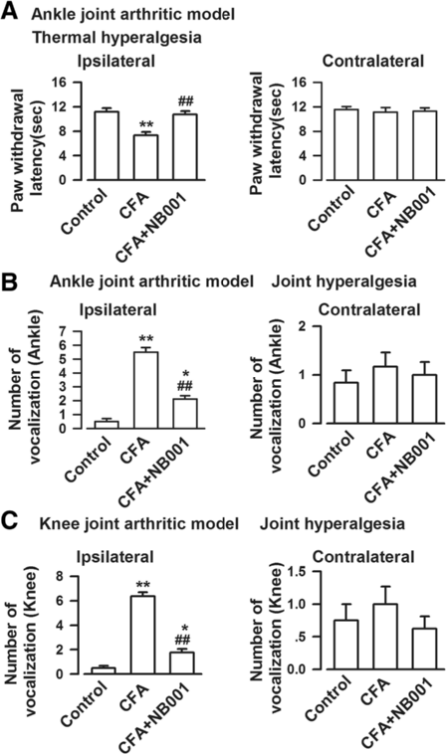
Effects of NB001 on thermal and joint hyperalgesia. Thermal hyperalgesia was examined on the last experimental day for ankle arthritis mice. **a** CFA caused thermal hyperalgesia in the ipsilateral hindpaw but not the contralateral hindpaw. NB001 administration (3 mg/kg, i.p.) significantly inhibit thermal hyperalgesia. **b** Joint hyperalgesia was assessed by the joint flexion test on the last experimental day and expressed as the numbers of vocalization caused by bending and extension of the joint (five times in each direction). The mice exhibited evident ankle joint hyperalgesia following CFA injection in the ipsilateral joint. **c** The mice exhibited markedly knee joint hyperalgesia following CFA injection in the ipsilateral joint. NB001 significantly (3 mg/kg, i.p.) attenuated both ankle (**b**) and knee joint hyperalgesia (**c**) indicted by reduction of vocalized numbers in joint flexion tests. *n* = 8 in each group. ^*^ *p* < 0.05, ^**^ *p* < 0.01 compared to the control group; ^##^ *p* < 0.01 compared to the CFA group

On the last day of the experiment (day 18), the thermal paw withdrawal latency of mouse models of ankle joint arthritis were measured. NB001 treatment (3 mg/kg) showed apparently higher thermal paw withdraw latency than saline treatment in CFA-injected mice (*F*_(2,21)_ = 26.555, *p* = 0.000, Dunnett’s test; Fig. 5a). Furthermore, the thermal latency in contralateral hindpaw was comparable among the three groups (*F*_(2,21)_ = 0.275, *p* = 0.762, LSD test).

### Effect of NB001 on joint hyperalgesia

Joint hyperalgesia was detected by recording the numbers of mice vocalization when bending and extension were applied to the joint. Figure. 5b, c show extremely low means for vocalization in the control mice. The numbers of vocalization in both mouse models of ipsilateral joint pain increased sharply in both mouse models (*F*_(2,21)_ = 123.141, *p* = 0.000, Dunnett’s test; Fig. 5b and *F*_(2,21)_ = 171.64, *p* = 0.000, LSD test; Fig. 5c). NB001 treatment (3 mg/kg) significantly decreased the number of vocalizations in both mouse models of ankle joint pain and knee joint pain. The numbers of vocalization among the groups in both models of contralateral joint pain were similar (*F*_(2,21)_ = 0.191, *p* = 0.828, LSD test; Fig. 5b and *F*_(2,21)_ = 0.653, *p* = 0.531, LSD test; Fig. 5c).

### NB001 exerted no effect on joint stiffness

**Fig. 6:**
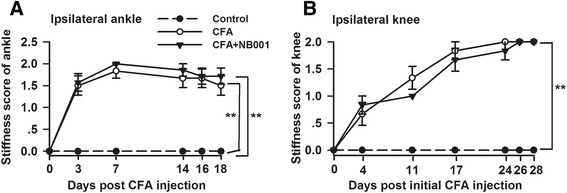
No effects of NB001 on CFA-induced joint stiffness. Joint stiffness was an indication of progressive disease development of arthritis. It was reflected by restriction of full range of joint movement. There was obvious restriction on the full-range joint movement in ipsilateral ankle (**a**) or knee (**b**) indicted by significant increase of joint stiffness scores. NB001 (3 mg/kg, i.p.) had no effects on joint stiffness. *n* = 8 in each group. ^**^ *p* < 0.01 compared to the control group

Joint stiffness was assessed by the bending and extension test and was used as an indicator in progressive arthritis development in adjuvant arthritis models [27]*.* No restriction was set on the full-range movement of joints in any mice on day 0, and the joints of the control mice showed no restriction throughout the whole experimental period (Fig. 6). The joint stiffness score increased sharply and remained at an increased level in the ipsilateral ankle following CFA injection (Fig. 6a). This score increased progressively in the ipsilateral knee following CFA injection (Fig. 6b). NB001 treatment (3 mg/kg) showed no evident influence on ankle or knee joint stiffness.

### NB001 showed no improvement on arthritis-induced joint damage

**Fig. 7:**
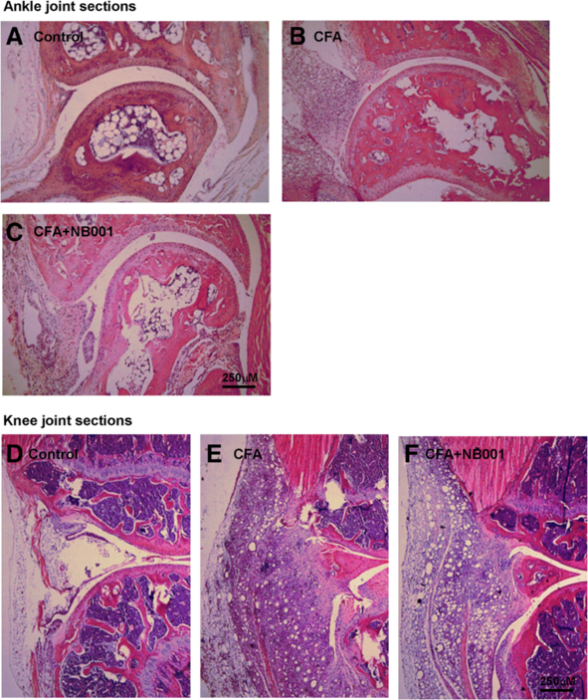
Histological staining of ankle and knee joints. The representative sections of ankle joint from control mice (**a**) and CFA-injection mice (**b**). CFA injection induced inflammatory cell infiltration, synovium hyperplasia, degeneration of the articular cartilage of the ankle joint. **c** NB001 treatment (3 mg/kg, i.p.) did not attenuate CFA-induced ankle joint structural destruction. The representative sections of knee joint from control mice (**d**) and CFA-injection mice (**e**). Four times knee intra-articular CFA injection induced abnormal structural changes of the knee joint, including bone, synovium, and meniscus. **f** NB001 treatment (3 mg/kg, i.p.) did not attenuate CFA-induced knee joint structural destruction

The histological evaluation of ankle and knee joint tissues was performed on day 18 and 28, respectively (Fig. 7). The histological architecture of the ankle joint showed abnormality in mice after the induction of ankle joint arthritis, as shown by inflammatory cell infiltration, synovial hyperplasia, and degeneration of the articular cartilage (Fig. 7b). Intra-articular CFA injection (4 times) caused abnormalities in knee joint structures, including pronounced synovial hyperplasia and inflammation, displacement of the meniscus, thickening of the joint capsule, and intra-articular fibrin accumulation (Fig. 6e). NB001 administration (3 mg/kg) for 5 days failed to evidently attenuate CFA-induced joint damages in both types of arthritic models (Fig.6c, f). These results demonstrated again that NB001 exerted no anti-inflammatory effects and failed to alleviate inflammation-induced joint damage.

## Discussion

Arthritis can affect people at any age, and its prevalence increases with age. Chronic joint pain, the most debilitating symptom of arthritis, prompts patients to seek medical help. However, few effective therapies exert no undesirable side effects [28]. Therefore, new therapies for alleviating arthritic joint pain need to be developed.

AC1 acts as a key molecule for triggering chronic-pain-related central plasticity. AC1 is considered indispensable for the induction of both spinal and ACC LTP, which is one of the possible cellular mechanisms underlying chronic pain [21]. Genetically, the knockout of AC1 reduced mice behavioral response to chronic pain caused by peripheral inflammation or nerve injury with the impaired LTP induction. Nociceptive responses were also remarkably inhibited in the persistent muscle pain model of AC1 knockout mice [25]. On the basis of these results, inhibiting AC1 activity may be a novel mechanism for chronic pain treatment [29]. As the first selective inhibitor of AC1, the analgesic effect of NB001 has been investigated in our previous studies. NB001 exhibited significant analgesic effects in the animal models of neuropathic pain, which was comparable to those produced by gabapentin [23]. Behavioral allodynia was also inhibited by NB001 in the animal models of inflammatory pain, with higher doses exerting greater inhibition [20].

Alleviating the joint pain of arthritic patients can increase functional recovery and improve quality of life. In this study, we investigated the analgesic role of NB001 on CFA-induced arthritic pain in two arthritis models. We demonstrated that NB001 produced antinociceptive effects in both mouse models of ankle joint pain and knee joint pain. However, NB001 failed to exhibit anti-inflammatory effects, as indicated by no attenuation of the related abnormal changes induced by inflammation.

Nociceptive articular pain originates locally in the joint at the site of inflammation or injury. CFA has been widely used to induce arthritic pain because of its ability to cause chronic inflammation in rodents [30] and produce a disease-like state that most closely resembles human arthritis [31]. CFA injection leads to T-lymphocytes and macrophage infiltration, which progress with joint swelling and synovial hypertrophy accompanied by significant reduction in joint cavity volume and severe osteolytic lesion [32, 33]. In our experiment, we first examined the effect of NB001 on CFA-induced joint inflammation. NB001 showed no obvious effects on joint edema caused by CFA injection. Joint stiffness results from long-term inflammation and indicates disease development together with abnormal mobility behavior in arthritic models [27]. Patients with arthritis also showed increased joint stiffness and decreased maximal amplitude of flexion and extension [34]. In the current study, mice exhibited apparent ankle or knee joint stiffness indicated by notable restriction of full-range joint movement following metatarsal or knee intra-articular CFA injection. Treatment with NB001 for 5 days exhibited no influence on the joint stiffness score. The effects of NB001 on joint swelling and stiffness implied that NB001 had no anti-inflammatory effect and could not prevent the progressive development of arthritis. This presumption was further demonstrated by the immunohistochemical analysis of a joint slice. CFA injection caused abnormal changes in both ankle joint and knee joint as indicated by synovial hypertrophy, inflammatory cell infiltration, degeneration of the articular cartilage, and thickening of the joint capsule. However, NB001 cannot improve inflammation-induced joint destruction.

For arthritis, the analgesic effect must be distinguished from an anti-inflammatory effect because analgesics alleviate pain but may not attenuate the progressive development of the disease [35, 36]. The analgesic effects of NB001 occurred in two types of CFA-induced arthritic pain model. Spontaneous pain (joint pain at rest) is an important behavioral symptom of arthritic joint pain, and the number of hindpaw flinches was recorded as the measure of spontaneous nociceptive behavior [6, 37]. CFA injections induced obvious spontaneous pain, as indicated by the increase in the number of hindpaw flinches. NB001 markedly inhibited spontaneous pain in our experiment. Furthermore, stimulus-evoked pain behavior was also examined to measure arthritic pain because it mimics the clinical condition of arthritic patients when the joint was moved [37]. Limb use scores during spontaneous movement and forced ambulation (rotarod) were used as the indexes of stimulus-evoked pain-related behavior according to previous research [38, 39]. NB001 administration markedly increased limb use scores in both mouse models of ankle and knee joint arthritis. These results showed that NB001 treatment inhibited spontaneous movement and evoked pain in adjuvant-induced arthritis.

Thermal hyperalgesia, mechanical allodynia, and joint hyperalgesia are the main secondary characteristics of arthritic pain [40, 41]. Under the condition of chronic arthritic pain, the threshold for nociceptor activation is lowered and the response to mechanical or thermal stimuli is amplified [42]. In our study, the mice showed significant and quick mechanical allodynia, thermal hypersensitivity, and joint hyperalgesia following CFA-induced ankle arthritis. However, knee intra-articular CFA injection led to a progressive reduction in mechanical threshold, and the mice exhibited apparent mechanical allodynia on day 17 after the initial CFA injection (3 days after the third CFA injection). These effects may be attributed to the fact that single knee joint intra-articular CFA injection could not adequately cause hindpaw mechanical hypersensitivity. NB001 treatment exhibited pronounced attenuation of behavioral sensitization, thus validating its analgesic effects on arthritic pain.

In conclusion, the results from our current study revealed that NB001 exerted analgesic effects on arthritic joint pain. Antinociception seemed unlikely to be associated with the anti-inflammatory effect because NB001 exhibited no anti-inflammatory action, as indicated by the lack of attenuation of joint edema, stiffness, and joint structural destruction caused by CFA. Combined with the results of previous studies with NB001, NB001 can be potentially used as a new analgesic.

## Methods

### Animals

All experiments were performed with adult male C57/BL6 mice aged from 8 to 10 weeks. Animals were housed under standard laboratory conditions (12 h light/12 h dark, temperature 22–26 °C, humidity 55–60 %) with food and water provided *ad libitum*. Animals were allowed to acclimate to laboratory environment for one week prior to the beginning of the experiment. All experiments were performed in accordance with protocols approved by Animal Care and Use Committee of the Fourth Military Medical University.

### CFA injection and NB001 administration

To induce arthritic pain of knee joint, CFA (10 μL, in 50 % saline) was injected into the left knee joint cavity of mice using a 30-gauge 1⁄2-in. needle every 7 days (on day 0, 7, 14, and 21) under isoflurane anesthesia [43]. For the ankle joint pain model, 10 μL CFA was injected into the left hind metatarsal footpad of mice on day 0*.* The same volume saline was injected into the left knee joint cavity or hind metatarsal footpad of the control mice. For the treatment, NB001 (3 mg/kg, in saline) was administrated intraperitoneally once a day from day 24 to day 28 for knee joint pain models or from day 14 to day 18 for ankle joint pain models. The first administration of NB001 was performed after the behavioral tests on day 14 or day 24. At the third and last administration of NB001, the behavioral tests were completed within 2.5 h after NB001 administration. The control and model (CFA) groups received intraperitoneal injection of saline at the same time point.

### Assessment of paw and knee joint edema

To quantify the effects of NB001 on the joint inflammation, the diameters across the ankle or knee joints were assessed using a vernier caliper before and after CFA injection at the different time points.

### Behavioral measurements of arthritic joint pain

All behavioral tests were performed during the day time and the animals were allowed to habituate to the laboratory room for at least 30 min before the tests. In all cases, behavioral analysis was performed by a trained observer blind to the experimental groups.

#### Evaluation of spontaneous pain and stimulus-evoked pain

Spontaneous pain and stimulus-evoked pain are two behavioral phenotype of arthritic joint pain and always used as the measurements of arthritis [44]. They reflect the clinical conditions of arthritic patients with joint pain at rest and after joint movement respectively [6, 37]. Hindpaw flinch is one indicator of spontaneous pain. Flinches were calculated as the number of times mice raised its hindpaw [44]. For the detection of flinching behavior, mice were placed in cylindric plexiglas chambers with the height of 30 cm and inside diameter of 20 cm for 30 min acclimation. The numbers of spontaneous flinch were recorded during 2 min. For the assessment of stimulus-evoked pain, the use of normal limb was detected and scored from 5 to 0 during spontaneous movement as previously reported [38, 39], 5 = normal use, 4 = partial limp, but not pronounced, 3 = pronounced limp, 2 = limp and guarding behavior, 1 = partial nonuse of limb in locomotor activity, 0 = totally lack of limb use. In addition, the limb-use was detected under forced ambulation condition. The mice were placed on a rotarod (Ji Liang Company, Shanghai, China) at a speed of 17 rounds/min for 5 min. Limb-use was scored: 4 = normal; 3 = limping; 2 = partial non-use of left hind limb; 1 = substantial non-use of left hind limb; 0 = non-use of left hind limb.

#### Mechanical allodynia test

Mice were put in individual plexiglas cages with a metal mesh floor to acclimate environment for 30 min before testing. Mechanical allodynia was detected with a set of von Frey filaments (Stoelting) and evaluated by hindpaw responsiveness to different stimulation. Mechanical pressure from the 1.65 filament (force, 0.008 g) was used to characterize the threshold stimulus based on the up-down strategy. The filaments were applied vertically to the dorsal surface of the hindpaw with sufficient force to cause slight bending for 6 s. Mechanical allodynia was detected five times with an interval of 10 min within stimulation. Positive responses include licking, biting, flinching, and brisk withdrawal of the hindpaw.

#### Thermal hyperalgesia test

Animals were placed in individual round container and allowed to adapt for 30 min before the experiment. Thermal hyperalgesia was evaluated by measuring the latency of paw withdrawal (PWL) in response to a radiant heat source [45] using a commercially available plantar analgesia instrument (BME410A, Institute of Biological Medicine, Academy of Medical Science, China). The heat source was turned off when the mice lifted the foot and the time from beginning to the ending of heat application was defined as the PWL. In order to prevent tissue damage resulted from long-time heat application, the heat source would be cut off automatically at 20 s even the mice did not lift the foot. The apparatus was modulated to give a paw withdrawal latency of approximately 10 s in naïve mice. Left paws were tested at 5 min intervals for a total of five trials. The mean PWL was obtained from the latter three stimuli [46].

#### Assessment of joint hyperalgesia

The joint hyperalgesia was measured according to the previous method [47] with little modification. Bending and extension (one after the other with a 5 s interval and five times in each direction) were applied to the ankle or knee joint within its limits of range of motion. The numbers of mice vocalized during each stimulus (the bending and extension) were recorded, and score 0 (no vocalization) or 1 (vocalization) was given depended on the results. Thus, for each mouse the vocalization score ranged from 0 to 10 at each joint [27, 48].

### Measurement of joint stiffness

The joint stiffness scoring was examined according to the previously reported method [32]. The mice were held stably, then the ankle or knee joint was bended and extended (once in each direction). The scores were given according to the following standard: score 2, there were restrictions of full range of joint movement in both bending and extension; score 1, there was restriction of movement of the joint only in one direction (bending or extension); score 0, there was no restriction in both direction [32].

### Histopathologic analysis

On the last experimental day, animals were anesthetized and sacrificed (day 28 after initial CFA injection into knee joint or day 18 after initial CFA injection into ankle joint) and the ipsilateral knee joints or ankle joints were collected. For collecting knee joints, the legs were cut through both the femur and the tibia. Then the specimens were immerged into 10 % neutral formalin to be post-fixed for 48 h. Subsequently, the fixed tissue was moved into decalcifying solution (4 M formic acid) for 35 days to make the bone completely demineralization. During this period, the decalcifying solution was changed once a week. The specimens were embedded in paraffin wax and cut into 5 μm thick sections, and stained with hematoxylin and eosin for microscopic assessment.

### Experimental procedure

The experimental procedure was summarized in Fig. 7. The upper one was experimental time course of ankle arthritis model and the lower one was that of knee arthritis model. All behavioral tests were performed prior to CFA injection and on day 3, 7, 14, 16, 18 for ankle arthritis or on day 4, 11, 17, 24, 26, 28 for knee arthritis after initial CFA injection. The first administration of NB001 was given after behavioral tests on day 14 for ankle joint arthritis models or on day 24 for knee joint arthritis models. During the treatment period, if there were behavioral measurements, they were finished within 2.5 h after NB001 or saline administration except it was specially mentioned.

### Data analysis

Data were presented as the mean and standard errors of the means (SEM). Statistical analysis of differences between two groups was performed by independent sample, two-tailed *t* test. Data of multiple groups were evaluated using one-way analysis of variance (ANOVA) for post hoc comparisons (SPSS 13.0). Data that passed the homogeneity test were analyzed by the one-way ANOVA least significant difference (LSD) test. Data that did not pass the homogeneity test were analyzed by the one-way ANOVA Dunnett’s test. In all cases, *p <* 0.05 was considered statistically significant.

## Abbreviations

AC1: Adenylyl cyclase subtype 1
ACC: Anterior cingulate cortex
ACSF: Artificial cerebrospinal fluid
cAMP: Cyclic adenosine monophosphate
CFA: Complete Freund’s Adjuvant
MPWT: Mechanical paw withdrawal threshold
LTP: Long-term potentiation
